# Surveying the Digital Competencies of Health Profession Educators at Ethiopian Higher Education Institutions

**DOI:** 10.4314/ejhs.v34i4.4

**Published:** 2024-07

**Authors:** Equlinet Misganaw Amare, Robel Tezera Zegeye, Awoke Giletew Wondie, Bekalu Assamnew Andargie

**Affiliations:** 1 Amref Health Africa; 2 Addis Ababa University, College of Medicine and Health Science, Addis Ababa, Ethiopia; 3 Debre-Tabor University, College of Health Science, Debre-Tabor, Ethiopia; 4 St. Paul Hospital Millennium Medical College, Addis Ababa, Ethiopia

**Keywords:** Digital learning, digital competency, digital skill competency, Ethiopia, health professional educators, higher education institution

## Abstract

**Background:**

The digital competency of health professional educators is crucial for institutions to develop digital skill policies and initiatives, plan professional development, and integrate technology into teaching practices. However, the development of policies, initiatives, and professional development programs related to digital skills is limited in low and middle-income countries due to a lack of empirical evidence. This study seeks to evaluate the digital competency of health professional educators in Ethiopian medical education.

**Methods:**

A survey was conducted at public Higher Education Institutions that provide health training programs in Ethiopia from April to September 2023 using the digital competency level assessment tool. Descriptive statistics were used to summarize the skill levels, and a chi-square test analysed the relationship between digital skills and various factors.

**Results:**

This study involved 498 health professional educators from 16 institutions. Most were male and taught clinical science. Over half worked in research institutions and held academic positions as assistant professors or above. About 10% were beginners in digital skills, with over half at an explorer level. Only a small percentage were experts. The majority had low digital skill competency, with only 7.2% showing high competency. There was no significant association between digital skill competency and factors like the type of institutions, program, course type or academic rank

**Conclusions:**

There is a notable lack of digital skill competency among faculty members, with the majority lacking expertise in this area. Educators require guidance, collaborative encouragement, and knowledge exchange to enhance their digital competencies and effectively harness technology in their teaching.

## Introduction

Digital learning has gained popularity in recent years in most higher education institutions (HEIs) worldwide due to its flexibility and accessibility (1-4). HEIs around the world are expanding digital learning to expand access to more students, meet increased demand for certain programs, extend the university's brand more broadly, innovate with teaching and learning, and potentially create additional income (4). Furthermore, the development and implementation of information communication technology (ICT) in the education sector has posed a challenge to the traditional learning environment by introducing new educational tools, including digital learning (5).

Digital learning approaches have gained momentum over the last decade within low-resource settings for the provision of education to healthcare providers. The use of digital learning for healthcare professionals in low- and middle-income countries (LMICs) is a relatively new concept. However, due to the outbreak of COVID-19, there has been a significant increase in the adoption of digital learning methods in these countries (6). The outbreak of COVID-19 especially changed the landscape of the educational systems and made the learning institutions shift from the traditional face-to-face to online teaching-learning modality (7-11).

Following the outbreak of COVID-19, HEIs in Ethiopia have made considerable investments in educational technology to adopt a digital learning approach. However, the effectiveness of digital learning in health professionals' education depends on various factors, including the design of the program, the quality of the learning materials, the level of institutional support, the acceptance of educational technology by faculty members (Health Professional Educators), and institutional readiness for digital learning (12-14). Additionally, the success of digital learning requires digital competency among learners and educators (14).

Digital competency has never been more important than it is now. Digital competency is the interest, attitude, and ability of individuals to use digital technology and communication tools appropriately to access, manage, analyze, and evaluate information, construct knowledge, and create and communicate with others (15).

Various studies have shown that the digital competency of instructors and technology, particularly in higher education institutions, is the most important aspect of digital learning (14, 16, 17). However, the introduction and utilization of digital learning for healthcare professionals in low- and middle-income countries (LMICs) is a relatively new concept. Studies have also revealed that educators lack competence in effectively utilizing digital learning technologies for teaching and learning purposes (18). The consequences of a lack of competence in digital skills among university teachers can have significant and far-reaching implications. Without proficiency in digital skills, teachers may encounter difficulties in accessing and utilizing online resources, academic databases, and educational platforms. Consequently, their ability to provide students with up-to-date information and diverse learning materials becomes limited.

Although several studies have reported competency gaps in the digital skills of educators, there is a lack of research and empirical evidence specifically in low and middle-income countries. This dearth of evidence can be attributed to the relatively underdeveloped state of digital learning, particularly in the education of health professionals. Assessing the digital competency of health professional educators is crucial for institutions to develop digital skill policies and initiatives, plan professional development, and integrate technology into teaching practices. These assessments will also help educators identify their current level of digital competence, recognize any gaps in their digital skills, and set goals for further development (19). Hence, the objective of this study is to assess the digital competency of health professional educators in using digital technologies for teaching in Ethiopian medical education.

## Methods and Materials

The study design involved an institution-based cross-sectional survey conducted between the months of April and September 2023. The study was conducted at 16 public HEIs that offer six key health training programs: Medicine, Nursing, Anaesthesia, Midwifery, Laboratory, and Pharmacy. HEIs in Ethiopia are classified into three categories: research institutions, universities of applied science, and comprehensive universities. Research universities concentrate on advanced postgraduate teaching, applied science universities on undergraduate courses, and comprehensive universities provide non-specific education. Over 5000 health professional educators worked in these HEIs (20). We used a single population proportion formula with 1.5 factors of design effect and proportional allocation to the sample size to select 577 for the study.

**Data collection tools and methods**: Data were collected from nine randomly selected public higher education institutions using the DigCompEdu digital competency assessment tool. DigCompEdu is a framework developed by the European Commission Joint Research Centre to help educators incorporate technology into their teaching. It is used as a reference tool for self-assessment, professional development, and the evaluation of digital competence (19), (21). The framework includes six areas of digital competence, and the study focused on teaching and learning. The framework further distinguishes six stages for competency levels in each area of digital skills. For each of the competency levels, level descriptors and competency statements or performance indicators are provided that allow educators to understand their level of competence and their specific development needs (Additional file 1).

Data collection took place from April to September 2023, with trained and experienced data collectors using the Open Data Kit (ODK) open-source Android app. This app is freely available for survey-based data-gathering purposes (22).

**Data analysis**: The ODK data were imported into SPSS V25 for cleaning and analysis. Descriptive statistics, such as frequency, percentage, and pie charts, were used to describe participant characteristics and summarize the digital skill competency level of Health professional educators for teaching. Additionally, a chi-square test for independence (with Yates Continuity Correction) was conducted to determine if there is a significant association between this high level of digital skill competency and various factors.

**Ethics approval and consent to participate**: This study was conducted with the approval of Jimma University, institute of Health Science Institutional Review Board (Ref.No-JUIH/IRB/266). The information sheet that explains the purpose of the research study, the procedure, the risks and benefits, and the confidentiality was read and explained to each study participant by the data collectors. Subsequently, the study participants were asked to provide their oral consent to participate in the research. It is worth noting that the participants willingly gave their oral consent to take part in this study, as it posed no significant risks or consequences

## Results

**Sample characteristics of the study participants**: This study involved the participation of 498 (response rate 86.3%) health professional educators from sixteen HEIs. The majority of the respondents were male, accounting for 459 (92.2%) participants, while 399 (80.1%) were involved in teaching clinical science courses. Additionally, more than half of the health professional educators worked in research institutions (53.6%) and held academic positions as assistant professors or above (52.4%). The respondents had a median teaching experience of 6 years and an average age of 33 years (Table 1).

**Table 1:** Characteristics of study participants (n=498)

Variables	Response	Frequency	Percentage
Sex	M	459	92.2
	F	39	7.8
	Total	498	100
Type of institution	Research Institution(n=8)	267	53.6
	Applied institution(n=3)	108	21.7
	Comprehensive Institutions(n=5)	123	24.7
	Total	498	100
Teaching unit/department	Clinical Science	399	80.1
	Non-Clinical science*	90	18.1
	Total	498	100
Academic qualification	First degree (BSc/MD)	18	3.6
	Second degree (MSc/MPH)	399	80.1
	Specialty & sub-speciality certificate (MD plus)	51	10.2
	Third Degree (PhD)	30	6.0
	Total	498	100
Academic rank	Lecturer	237	47.6
	Assistant Professor and above	261	52.4
	Total	498	100
	33.6	Min	Max
Mean Age of Health professional educators		23	51
Median Teaching Experience in HEIs in Years	8	1	25

* Nonclinical science course includes all basic science (anatomy, physiology, biochemistry, and public health science)

**Health professional educators' digital skill competency**: Overall, the statistics reveal that 9 out of 10 Health professional educators (92.8%) possess a digital skill competency level below expertise. Conversely, only a small percentage (7.2%) of Health professional educators demonstrate a high level of competency, indicating expertise and above, in utilizing digital technologies for teaching (Figure 1).

When it comes to the utilization of digital learning technologies for instruction, one in ten Health professional educators (10.2%) were novices in using digital skills for teaching, while more than half of Health professional educators had a competency level of explorer in utilizing digital technologies for teaching. Approximately 29.5% of Health professional educators were integrators in terms of competency level. Only a few Health professional educators (less than 5%) demonstrated expert competency in utilizing digital technologies for teaching (Table 2).

**Table 2:** Health professional educators categorized by their digital skill competency level in the use of digital technologies for teaching

Digital skill competency level	n(%)				

	Used for instruction	Used for student guidance and support	Used for collaborative learning	Used for foster SDL	Mean
Level 1: Novice	51(10.2)	75(15.1)	174 (34.9)	126 (25.3)	21.38
Level 2: Explorer	258(51.8)	270(54.2)	252(50.6)	294(59.0)	53.9
Level 3: Integrator	147(29.5)	111(22.3)	48(9.6)	42(8.4)	17.45
Level 4: Expert	15(3.0)	30(6.0)	6(1.2)	27(5.4)	3.9
Level 5: Leader	21(4.2)	9(1.8)	19(3.6)	9(1.8)	2.85
Level 6 Pioneer	6(1.2)	3(0.6)	0(0.0)	0(0)	0.45
Total	498(100)	498(100)	498(100)	498(100)	

Regarding the utilization of digital learning technologies for student guidance and support, the majority of Health professional educators (54.2%) were classified as explorers in terms of their competency level in digital skills. They were followed by integrators (22.3%) and novices (15.1%) in their digital skill competency level (Table 2). The utilization of digital learning technologies for collaborative learning, an overwhelming majority of health professional educators, constituting more than 75%, exhibited a novice or explorer level of competency. Conversely, a mere 1% of health professional educators reported themselves as experts in this domain. In the realm of digital learning technologies that promote self-directed learning, it is intriguing to note that more than one-fourth (25.3%) of faculty members fall into the category of novices, while 59% fall into the category of explorers. Only 8.4% of faculty members and professionals are considered experts in the use of digital learning technologies to foster self-directed learning (Table 2).

A chi-square test for independence (with Yates Continuity Correction) was conducted to determine if there is a significant connection between this high level of digital skill competency and various factors. The findings from the test indicate that there is no significant association between a high level of digital skills competency and the specific teaching environment (χ2 (2, n = 498) = 5.727, p = 0.06). Similarly, the type of course being taught does not appear to have a significant impact on digital skill competency (χ2 (1, n = 489) = 0.85, p = 0.77). Furthermore, academic rank does not seem to be associated with a higher level of digital skill competency (χ2 (1, n = 498) = 2.1, p = 0.145) (Table 3).

**Table 3:** Relationship between the digital skill competency of Health professional educators in using digital technologies and their demographic factors

	The proportion of Health professional educators who possess digital skill competency at the Expertise level and higher.				

Variable		Yes	No	Total	P value
		Number(%)	Number(%)	Number(%)	
Type of university	Research	27(64.3)	240(52.6)	267(53.6)	0.06
	Applied	3(7.1)	105(23.0)	108(21.7)	
	Comprehensive	12(28.6)	111(24.3)	123(24.7)	
	Total	42 (8.4)	456(91.6)	498(100)	
Teaching unit	Clinical	33(84.6)	366(81.3)	399(81.6)	0.770
	Nonclinical	6(15.4)	84(18.7)	90(18.4)	
	Total	39 (100)	450(100)	489(100)	
Academic rank	Lecture	15(35.7))	222(48.7)		0.145
	Assistant Professor and above	15(35.7)	234(51.3)		
	Total	237(47.6)	261(52.4)	498(100)	

## Discussion

The effectiveness of digital learning in medical education highlights the need for both learners and educators to possess digital competency (14). However, the underdeveloped state of digital learning in low and middle-income countries, particularly in the education of health professionals, has resulted in a dearth of evidence regarding the digital competency of health professional educators in higher education institutions. This study represents the first of its kind to assess the digital competency of health professional educators in Ethiopian medical education.

Medical educators' digital competency plays a crucial role in improving the quality of education and patient outcomes. Medical educators who are digitally literate can effectively curate and share these resources with their students, ensuring they have access to the most current information. This improves the quality of education by promoting evidence-based practice and keeping students informed about the latest advancements in medicine (13).

Digital competency is about more than just using computers. To become digitally literate, educators need to develop a range of skills. They need to be able to use technology to search for and create content, solve problems and innovate. They need to be able to connect and communicate effectively online, learn, collaborate with peers, and discover and share new information (23).

The findings in this study revealed that approximately 21.3%, 53.9% and 17.4% of FM digital skill competency levels fall into the categories of novice, explorer and integrator, respectively. The novice level of digital skill competency implies that Health professional educators are aware of the potential of digital technologies. However, they have had very little contact with digital technologies. Novice needs guidance and encouragement to expand their repertoire and to apply their existing digital competence in the pedagogical realm (19). Explorers are aware of the potential of digital technologies and are interested in exploring them to enhance pedagogical and professional practice. They have started using digital technologies in some areas of digital competence, without, however, following a comprehensive or consistent approach. Explorers need encouragement, insight, and inspiration (19).

Integrators experiment with digital technologies in a variety of contexts and for a range of purposes, integrating them into many of their practices. They creatively use them to enhance diverse aspects of their professional engagement. They are eager to expand their repertoire of practices. They are, however, still working on understanding which tools work best in which situations and fitting digital technologies to pedagogic strategies and methods. Integrators just need more time for experimentation and reflection, complemented by collaborative encouragement and knowledge exchange to become experts (19).

Overall, the statistics reveal that 9 out of 10 health professional educators (92.8%) possess a digital skill competency level below expertise. Conversely, only a small percentage (7.2%) of health professional educators demonstrate a high level of competency, indicating expertise and above, in utilizing digital technologies for teaching. Experts use a range of digital technologies confidently, creatively, and critically to enhance their professional activities. They purposefully select digital technologies for particular situations and try to understand the benefits and drawbacks of different digital strategies. They are curious and open to new ideas, knowing that there are many things they have not tried out yet. They use experimentation as a means of expanding, structuring and consolidating their repertoire of strategies (19).

These findings highlight a significant gap in digital skill competency among Health professional educators, with the majority falling short of expertise. Imagine a scenario where a teacher in higher education institutions is unable to navigate online resources effectively. They may miss out on valuable research articles, scholarly publications, or even interactive learning tools that could greatly enhance their teaching methods. This deficiency in digital skills not only hampers their own professional growth but also deprives students of the opportunity to engage with cutting-edge information and materials (24). Furthermore, the consequences of inadequate digital skills extend beyond the classroom. In an era where remote learning and online education have become increasingly prevalent, teachers must be equipped with the necessary tools to effectively engage students in virtual environments. Without digital competence, teachers may find it challenging to create interactive online lessons, facilitate virtual discussions, or even provide timely feedback to students. As a result, the quality of education suffers, and students may not receive the support and guidance they need to succeed in their academic endeavours (24, 25).

This lack of competence in utilizing digital learning technologies for teaching and learning purposes among health professional's educators can be attributed to several factors. Firstly, limited access to technology and internet connectivity hinders the effective implementation of digital learning initiatives. Many health professional's educators in these countries may not have access to personal computers or reliable internet connections, making it difficult for them to engage with online learning platforms. Additionally, there may be a lack of awareness and training opportunities for healthcare educators on how to effectively integrate digital learning into their teaching methods. Furthermore, cultural barriers and resistance to change within educational institutions can also impede the adoption of digital learning. Traditional teaching methods may be deeply ingrained in the education system, making it challenging for educators to embrace new technologies and adapt their teaching approaches accordingly (24, 26). This highlights the need for further training and support to enhance health professional educator' digital skills and ensure that they are equipped to effectively integrate technology into their teaching practices.

It is worth noting that the lack of association between digital skill competency and factors such as types of university, teaching units, and academic rank suggests that these variables do not necessarily influence an individual's level of digital skill competency. This finding suggests that health professional educator can reap the benefits of enhancing their digital skills, regardless of their teaching environment, course type, or academic rank. Moreover, it indicates that the development of digital skills may be linked to individual motivation, commitment, and practice. Thus, it implies that anyone, irrespective of their academic rank or teaching context, can improve their digital competency through personal dedication and consistent practice.

Our results align with previous research conducted by Smith et al. (2018), who emphasized the importance of individual effort in acquiring digital skills. They argued that individuals who possess a strong intrinsic motivation to learn and improve their digital abilities are more likely to succeed in developing expertise in this domain (24, 25). Furthermore, our findings support the notion that continuous practice plays a crucial role in achieving high levels of digital skill competency. As highlighted by Johnson (27), deliberate practice is essential (28).

The findings of this study play a crucial role in helping educators identify their current level of digital competence. Through this study, we aspire to not only enhance our understanding of the digital skills landscape among health professional educator but also contribute to the development of effective strategies for improving their competency. These findings provide valuable insights into the strengths and weaknesses of educators' digital skills, enabling them to pinpoint specific areas for improvement. By recognizing their digital skill gaps, educators can then set realistic goals for further development. Institutions can utilize these findings to tailor professional development programs that address the specific needs of their educators. This personalized approach ensures that educators receive targeted training and support, maximizing their potential to effectively utilize technology in the classroom. Furthermore, these findings allow institutions to allocate resources efficiently.

This study is the first of its kind to assess the level of digital skill competency among health professional educator in higher education institutions. Additionally, it encompasses a significant number of study participants, representing nearly all levels of institutions. However, despite the comprehensive data collection from a wide-ranging pool of health professional educators, the findings hold significant relevance on an individual basis. The study is subject to several limitations. Firstly, its sectional design doesn't allow for establishing a causal relationship between exposures and outcome variables, relying heavily on self-reported data. This opens the door to potential biases such as social desirability or subjective interpretations. Furthermore, it's crucial to acknowledge that these findings may not be broadly applicable to the entire community of health professional educators.

Even though experts' level of digital competency skills is crucial to the success of educational institutions in driving innovation in practice, recent findings have shed light on a significant deficiency in digital skill competency among health professional educators. The majority of them lack expertise in this area. The findings also indicated that there is no significant association between a high level of digital skills competency and the specific teaching environment, the type of course being taught, and the academic rank of health professional educators.

Health professional educators require guidance, collaborative encouragement, and knowledge exchange to enhance their digital competencies and effectively harness technology in their teaching. Addressing this gap is essential for empowering health professional educators to leverage the full potential of digital tools in their educational practices. The results of this study will offer valuable insights for institutions looking to improve their digital skills policies and initiatives. These findings will play a crucial role in developing professional development programs and integrating technology into teaching practices effectively. Additionally, the findings will assist educators in assessing their digital proficiency, identifying areas for improvement, and setting goals for continuous growth and advancement.
